# *Lactiplantibacillus plantarum* peptides and probiotics from peanut butter enhance antimicrobial activity and gut microbiota modulation

**DOI:** 10.3389/fnut.2026.1927533

**Published:** 2026-09-17

**Authors:** Mansour Alblaji, Shaaban H. Moussa

**Affiliations:** 1 Department of Basic Health Sciences, College of Applied Medical Sciences, Qassim University, Buraydah, Saudi Arabia; 2 Department of Biological Sciences, Faculty of Science and Humanities, Shaqra University, Ad-Dawadimi, Saudi Arabia

**Keywords:** antimicrobial synergy, gastrointestinal stability, *Lactiplantibacillus plantarum*, peanut peptides, short-chain fatty acids, therapeutic nutrition

## Abstract

**Background:**

Fermented plant matrices with probiotics show antimicrobial and gut health potential. Still, the quantitative links between peanut fermentation, peptide–probiotic synergy, digestive survival, and microbiota modulation remain unclear.

**Objective:**

We optimized bioactive peptide production from reduced-fat (33%) and full-fat (50%) peanut butter fermented with *Lactiplantibacillus plantarum* ATCC 14917 for 0–72 h using a full factorial design.

**Methods:**

The < 3 kDa peptide fraction was combined with *Lactobacillus* acidophilus ATCC 4356 and *Bifidobacterium bifidum* ATCC 29521 at sub-inhibitory levels. Antimicrobial activity against *Salmonella enterica* serovar Typhimurium, *Listeria monocytogenes*, and *Clostridioides difficile* was tested by agar diffusion, MIC, MBC, and checkerboard assays. Gastrointestinal stability followed the INFOGEST protocol, and colonic fermentation used fecal inoculum from three healthy donors to track microbiota and short-chain fatty acids (SCFAs).

**Results:**

At 72 h of fermentation, reduced-fat matrix reached 61.3% hydrolysis and 9.45 log10 CFU/mL, which was 16% above full-fat. The combination treatment (5 mg/mL peptides plus 4.5 × 104 CFU/mL probiotics) lowered *L. monocytogenes* MIC by 75% to 1.25 mg/mL, with a fractional inhibitory concentration (FIC) index of 0.50 indicating synergy. During simulated gastrointestinal digestion, the combination maintained 87.1% bacterial survival and 85.8% peptide retention. In colonic fermentation, the combination raised *Bifidobacterium* to 8.93 log_10_ CFU/mL and *Lactiplantibacillus* to 8.71 log_10_ CFU/mL, dropped *Clostridium* to 3.31 log_10_ CFU/mL, increased the B/C ratio to 2.70, and boosted total SCFAs by 467% to 46.5 μmol/mL.

**Conclusion:**

*L. plantarum* fermentation of reduced-fat peanut generates peptides that, with probiotics, give synergistic antimicrobial effects, better GI stability, and microbiota shifts toward higher SCFAs. This supports peanut-based synbiotic foods.

## Introduction

Foodborne pathogens and spreading antibiotic resistance have pushed food safety to the top of the global agenda, forcing researchers to look beyond synthetic antibiotics and chemical preservatives. As a result, bioactive antimicrobial peptides from food proteins have moved to the front line as candidate solutions. What makes them attractive is how they act: they rupture bacterial membranes and block intracellular targets, and on top of that they come from natural sources, carry a solid safety record, and are less likely to drive resistance than conventional drugs (1–3). The story does not end at killing pathogens directly; more data now show these peptides also reshape gut microbiota, boost immune modulation, and provide antioxidant activity, which ties them to both chronic disease prevention and food preservation (1, 4, 5).

Lactic acid bacteria (LAB) are the main factories for making peptides inside foods, and *Lactiplantibacillus plantarum* matters here because it is Generally Recognized As Safe (GRAS) and has strong proteolytic power (1, 2). Work on fermented milk shows that low molecular weight peptide fractions (< 3 kDa) hit *Escherichia coli*, *Staphylococcus aureus*, *Listeria monocytogenes*, and *Salmonella* hard, largely by permeabilising membranes (6–8). Genome surveys have also picked up several bacteriocin and ribosomally synthesized and post-translationally modified peptide (RiPP) gene clusters in *L. plantarum* strains, giving molecular proof they can synthesize antimicrobial peptides and postbiotics (9, 10).

The catch is that peptides and probiotics do not always survive the gut; viability and structure drop under gastric and intestinal conditions, so lab activity does not always translate. To deal with this, standardized *in vitro* digestion protocols such as INFOGEST are now used to mimic oral, gastric, and intestinal transit and test stability and survival (11–13). Across these models the food matrix keeps coming up as decisive; lipid- and protein-rich carriers and encapsulation lift survival of *Lactiplantibacillus* and *Bifidobacterium* and keep peptides intact (12–15).

Beyond just getting through digestion, fermented foods shift gut microbiota and short-chain fatty acid (SCFA) patterns, raising beneficial taxa and pushing up acetate, propionate, and butyrate (16–18). Probiotic-driven changes have also been tied to weaker inflammatory responses (19). In the context of fermented foods, the peptide-probiotic formulation shares mechanistic features with Type II sourdough fermentation, where prolonged fermentation with selected lactic acid bacteria enhances bioavailability of bioactive compounds and modulates gut microbiota (20). Similarly, low-FODMAP fermented foods have gained attention for their ability to reduce gastrointestinal symptoms while maintaining microbiome diversity (21). The techno-functional properties and biosafety of such formulations meet criteria proposed for novel fermented starters (22).

Even with that, big gaps remain: production is costly, *in vivo* data are thin, and many peptides lose activity in complex foods. More importantly, very few studies have looked directly at how bioactive peptides, probiotics, and food matrices interact together under digestion and colonic fermentation that reflect human physiology (1, 3).

For this reason, we focused on *L. plantarum*-fermented peanut butter as a dual delivery platform. Specifically, we tested whether combining fermented peanut-derived peptides with probiotic strains produces synergistic antimicrobial activity against *Salmonella enterica* serovar Typhimurium, *Listeria monocytogenes*, and *Clostridioides difficile*, whether viability holds during simulated gastrointestinal digestion, and whether the mix shifts human gut microbiota composition and SCFA production during *in vitro* colonic fermentation. This setup gives a mechanism-driven read on peptide-probiotic systems within food matrices and what they might actually do in the human gut.

## Materials and methods

### Study location, ethical approval, and experimental design

This study was carried out at Shaqra University, Shaqra, Saudi Arabia, from January 2024 to June 2025. The Institutional Review Board approved the use of human fecal samples, and all donors provided written informed consent before collection. We recruited three healthy adults aged 25–35 years with a body mass index (BMI) of 18.5–24.9 kg/m^2^ to reduce inter-individual variation in colonic fermentation, following Payne et al. (23). Participants had no antibiotic or probiotic use for 3 months, no gastrointestinal disorders, and followed a non-vegan diet.

The work followed a three-phase sequential design as shown in Figure 1. We moved from peanut butter fermentation and peptide extraction to simulated digestion and then to *in vitro* colonic fermentation. All experiments were done in three biological replicates, and data are shown as mean ± standard error.

**Figure 1:**
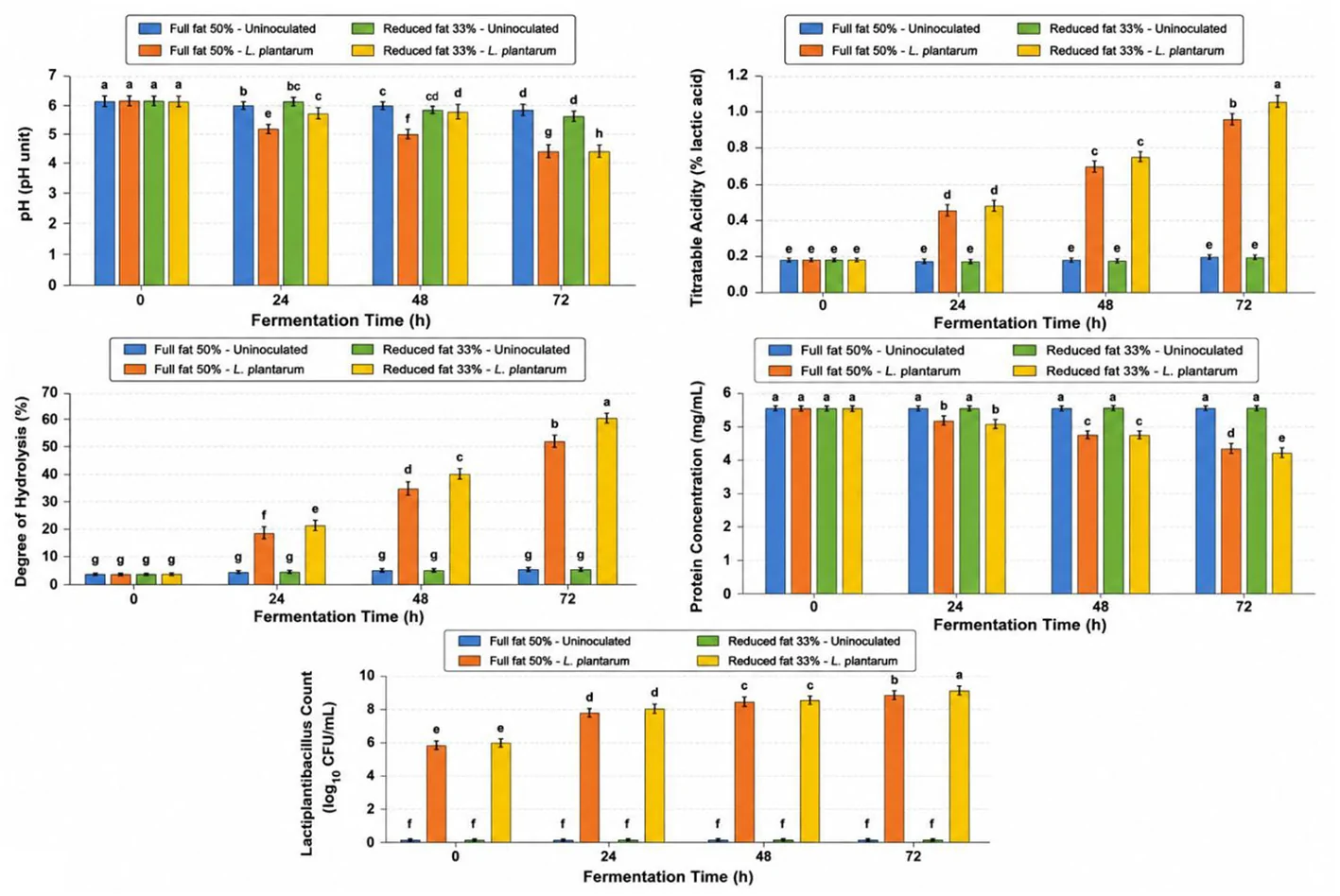
Schematic workflow of the three-phase experimental design. The workflow figure presents a simplified diagram showing Phase 1 (Fermentation and Peptide Extraction), Phase 2 (Antimicrobial and Synergy Assays), and Phase 3 (Gastrointestinal Digestion and Colonic Fermentation) with sample flow.

### Materials, bacterial strains, and culture conditions

Commercial smooth peanut butter was purchased locally in two matrices: full-fat (50% fat) and reduced-fat (33% fat). Proximate analysis showed the full-fat matrix contained 50.0 ± 0.5% fat, 25.0 ± 0.3% protein, and 20.0 ± 0.4% carbohydrate, whereas the reduced-fat matrix contained 33.0 ± 0.4% fat, 28.0 ± 0.3% protein, and 34.0 ± 0.5% carbohydrate (mean ± SD, *n* = 3; AOAC methods).

For peptide production we used *Lactiplantibacillus plantarum* ATCC 14917. The probiotic strains were *Lactobacillus* acidophilus ATCC 4356 and *Bifidobacterium bifidum* ATCC 29521. The designation *Lactobacillus acidophilus* is used throughout, consistent with current taxonomic nomenclature (24). Pathogens tested included *Salmonella enterica* serovar Typhimurium ATCC 14028, *Listeria monocytogenes* ATCC 19115, and *Clostridioides difficile* ATCC 9689. All strains were obtained from the American Type Culture Collection (ATCC) and stored at −80 C in de Man Rogosa Sharpe (MRS) broth with 20% glycerol, as described by De Man et al. (25).

Anaerobic conditions were maintained using anaerobic jars (GasPak™ EZ, Becton Dickinson, USA) with gas-generating sachets and verified with indicator strips (Oxoid, UK). For liquid cultures, Hungate tubes were flushed with 100% N_2_ gas for 5 min and sealed with butyl rubber stoppers. Culture conditions were strain-dependent. *L. plantarum* ATCC 14917 and *L. acidophilus* ATCC 4356 were grown in MRS broth anaerobically at 37 C for 24 h (25). *B. bifidum* ATCC 29521 required MRS broth supplemented with 0.05% cysteine and was incubated anaerobically at 37 C for 48 h (26). The two aerobic pathogens, *S. Typhimurium* ATCC 14028 and *L. monocytogenes* ATCC 19115, were cultured in Tryptic Soy Broth (TSB) aerobically at 37 C for 18 h. *C. difficile* ATCC 9689 was grown in Reinforced Clostridial Medium (RCM) anaerobically at 37 C for 48 h (27).

The study used a range of culture media and reagents: MRS agar, TSB, RCM agar, XLD agar, PALCAM agar, CCFA agar, Bifidobacterium selective agar (Oxoid, UK; HiMedia, India); pepsin (≥250 U/mg), pancreatin (8 × USP), bile salts, OPA reagent, BSA, gentamicin (10 μg), ampicillin (10 μg), metronidazole (5 μg) (Sigma-Aldrich, USA); Amicon Ultra-15 3 kDa filters (Merck Millipore, USA); and QIAamp PowerFecal Pro DNA Kit (Qiagen, Germany).

### Phase 1: production and characterization of bioactive peptides

#### Experimental design

We applied a three-factor full factorial design to cover fat type at two levels (full-fat 50% and reduced-fat 33%), inoculation at two levels (*L. plantarum* ATCC 14917 and an uninoculated control), and fermentation time at four levels (0, 24, 48, and 72 h). This setup produced 16 treatment combinations. We chose this design to capture main effects and interactions without unnecessary runs (28).

#### Fermentation and peptide extraction

Peanut butter homogenates were prepared at 10% w/v in sterile phosphate-buffered saline (PBS, pH 7.2). *L. plantarum* was activated by two successive transfers in MRS broth at 37 C for 24 h under anaerobic conditions. Cells were harvested at 4,000 × g for 10 min at 4 C, washed twice with PBS, and resuspended to 8.0 log_10_ CFU/mL using McFarland standard 2.0, which we verified by plate count on MRS agar with the pour plate method (29, 30).

Homogenates received a 1% v/v inoculum to reach ~6.0 log_10_ CFU/mL, while controls received sterile PBS. Fermentation was performed at 37 C for 72 h with shaking at 150 rpm under microaerobic conditions using cotton-plugged flasks to allow limited air exchange, which supports *L. plantarum* growth and is more practical for scale-up than strict anaerobiosis. We sampled at 0, 24, 48, and 72 h for kinetic analysis.

After 72 h, samples were centrifuged at 10,000 × g for 15 min at 4 C, then the supernatant was filtered through a 0.45 μm membrane and ultrafiltered with Amicon Ultra-15 3 kDa filters at 4,000 × g, 4 C. The permeate containing peptides < 3 kDa was lyophilised and stored at −80 C for Phase 2, following peptide isolation protocols from Korhonen and Pihlanto (30).

#### Analytical methods

In Phase 1 we tracked how fat level, inoculation, and fermentation time shaped the peptide profile by measuring five dependent variables: pH (calibrated pH meter), titratable acidity (% lactic acid by titration to pH 8.3; AOAC Method 947.05), degree of hydrolysis (%) [OPA method at 340 nm; (31)], protein concentration (mg/mL) [Bradford assay with BSA standard; (32)], and *Lactiplantibacillus* count (log_10_ CFU/mL) [plate count on MRS agar, anaerobic, 37 C, 48 h; (25)].

### Phase 2: antimicrobial and synergistic activity assessment

#### Experimental design

We used a two-way factorial design in Phase 2 with ‘Treatment Type' at three levels and ‘Pathogen Type' at three levels. Treatments included peptides at 10 mg/mL, probiotics at 9.0 log_10_ CFU/mL, a combination of peptides (5 mg/mL) + probiotics (4.5 × 10^4^ CFU/mL; a 10^4.5^ dilution of the stock), and a Negative Control (PBS). The probiotic dose of 4.5 × 10^4^ CFU/mL (i.e., 10^4.5^) was chosen as a sub-inhibitory level; this concentration alone caused no visible inhibition but was expected to synergise with peptides. For statistical analysis, we ran two-way ANOVA on MIC, MBC, and zone of inhibition using the three treatment levels, and we excluded the Negative Control from models due to zero variance, reporting it descriptively instead. Pathogens were *S. Typhimurium* ATCC 14028, *L. monocytogenes* ATCC 19115, and *C. difficile* ATCC 9689. Although these pathogens differ biologically, we treated them as categorical levels of the fixed factor ‘Pathogen Type' because responses were measured under identical conditions, and residuals met normality and homoscedasticity assumptions by Shapiro-Wilk and Levene's tests (*p* > 0.05). Concentration ranges followed synergy testing guidelines (33).

#### Antimicrobial and synergy assays

Agar well diffusion and broth microdilution were used to determine antimicrobial activity, as described by Wiegand et al. (34) and Balouiri et al. (35). For agar well diffusion, 50 μL of each sample was placed into 6 mm wells on Mueller-Hinton agar seeded with ~10^6^ CFU/mL. After incubation, inhibition zones were measured with a digital caliper and reported as zone of inhibition (mm). This variable was included in the two-way ANOVA.

For minimum inhibitory concentration (MIC, mg/mL) and minimum bactericidal concentration (MBC, mg/mL), 2-fold serial dilutions were prepared in 96-well plates and inoculated with 5 × 10^5^ CFU/mL. MIC was defined as the lowest concentration with no visible growth. MBC was determined by subculturing 10 μL from clear wells onto fresh agar, and was defined as a ≥3 log_10_ reduction (34). Both MIC and MBC were included in the two-way ANOVA; the negative control was excluded from the statistical model.

Synergy between peptides and probiotics was assessed using a checkerboard assay in 96-well plates with varying ratios of the two agents. The fractional inhibitory concentration (FIC) index was calculated for the combination treatment only as FIC = FIC (peptide) + FIC (probiotic). An FIC index ≤ 0.5 indicated synergy, and >0.5 to ≤ 1.0 indicated additivity (33). The FIC index was analyzed descriptively and was not included in the ANOVA because it applied only to the combination treatment.

### Phase 3: simulated gastrointestinal digestion and colonic fermentation

#### Phase 3A: simulated gastrointestinal digestion

We applied the INFOGEST static *in vitro* digestion protocol with minor modifications, following Brodkorb et al. (36). Three treatments were evaluated: probiotics alone at 9.0 log10 CFU/mL, peptides alone at 5.0 mg/mL, and a combination of peptides (5.0 mg/mL) plus probiotics (9.0 log_10_ CFU/mL). A PBS control was included for reference.

The digestion sequence consisted of three phases: oral (2 min, pH 7.0), gastric (2 h, pH 3.0, pepsin 2,000 U/mL), and intestinal (2 h, pH 7.0, pancreatin 100 U/mL, bile salts 10 mM). Samples were collected after each phase to monitor bacterial viability and peptide content.

Five dependent variables were tracked: final bacterial count (log_10_ CFU/mL) on MRS agar; bacterial survival (%) = (Final CFU/mL / Initial CFU/mL) × 100; final peptide concentration (mg/mL) by Bradford assay; peptide retention (%) = (Final concentration / Initial concentration) × 100; and final pH (pH meter). Initial bacterial count was fixed at 9.0 log_10_ CFU/mL and initial peptide concentration was fixed at 5.0 mg/mL across all treatments and phases; these fixed variables were excluded from ANOVA models.

#### Phase 3B: colonic fermentation

We ran batch colonic fermentations with six treatment groups sampled at 0, 24, and 48 h: Uninoculated Control (fecal inoculum only), Solvent Control (fecal inoculum + PBS), Peptides (fecal inoculum + 5.0 mg/mL peptides), Probiotics (fecal inoculum + 9.0 log_10_ CFU/mL probiotics), Combination Treatment (fecal inoculum + peptides + probiotics), and Media Control (YCFA medium only). Fermentation was carried out in YCFA medium under anaerobic conditions at 37 C for 48 h, following Payne et al. (23). Fecal material was collected from the three donors within 2 h of defecation, transported in sterile anaerobic bags (BD GasPak™), and processed immediately. Samples were diluted 1:10 (w/v) in pre-reduced PBS (pH 7.2) with 0.05% L-cysteine HCl, homogenized for 2 min, and filtered through sterile gauze. The filtrate was centrifuged at 500 × g for 5 min to remove debris, and the supernatant containing the microbial fraction was used as inoculum (10% v/v) in YCFA medium. All handling was done in an anaerobic chamber (Coy Laboratory Products, USA) with 85% N_2_, 10% CO_2_, 5% H_2_.

#### Phase 3B: analytical methods

Nine dependent variables were analyzed: *Bifidobacterium* count (log_10_ CFU/mL) on Bifidobacterium selective agar (26); *Lactiplantibacillus* count (log_10_ CFU/mL) on MRS agar (25); *Clostridium* count (log10 CFU/mL) on CCFA agar (27); B/C ratio (calculated); total SCFAs, acetic acid, propionic acid, butyric acid (μmol/mL) by GC-FID (37); and Shannon index from 16S rRNA sequencing [QIIME 2; (38)]. Three additional variables—pH, Simpson index, and observed features—were measured but reported descriptively only because they did not meet parametric assumptions.

For GC-FID, we used a DB-FFAP column (30 m × 0.25 mm × 0.25 μm) with injector at 250 C; oven program: 80 C for 1 min, increased to 200 C at 10 C/min, held 5 min; isobutyric acid as internal standard. For microbiome analysis, DNA was extracted using QIAamp PowerFecal Pro Kit, V3-V4 region amplified (341F/805R), sequenced on Illumina MiSeq (2 × 300 bp), processed in QIIME 2 with DADA2, taxonomy assigned against SILVA 138.1.

### Statistical analysis

Data were analyzed using SPSS version 28.0 and R version 4.2.2. We checked normality with Shapiro-Wilk and homogeneity of variance with Levene's test. In Phase 1, three-way ANOVA was used. In Phase 2, two-way ANOVA was applied to three dependent variables using treatment type at three levels and pathogen type at three levels; the Negative Control was excluded from MIC and MBC models due to zero variance, and the FIC index was reported descriptively. For Phase 3A, two-way ANOVA was performed on five dependent variables; initial bacterial count and initial peptide concentration were fixed and excluded. In Phase 3B, two-way ANOVA was run for nine variables; pH, Simpson index, and observed features were reported descriptively. Tukey's HSD *post-hoc* test was used for multiple comparisons at *p* < 0.05.

## Results

### Fermentation parameters

The three-way ANOVA (Supplementary Table S1) explained most of the variability (R^2^ = 0.975–0.982) across pH, titratable acidity, degree of hydrolysis, protein concentration, and *Lactiplantibacillus* count. Fermentation time was the dominant factor (MS = 2,251.3 for degree of hydrolysis, 41.54 for bacterial count, *p* < 0.001). As time advanced, degree of hydrolysis, titratable acidity, protein concentration, and bacterial count all rose; pH declined steadily. Inoculation with *L. plantarum* likewise produced highly significant effects (*p* < 0.001) for all variables, with higher hydrolysis, acidity, protein depletion, and bacterial counts, and lower pH. Fat type was significant for hydrolysis (*p* < 0.01), pH (*p* < 0.01), protein concentration (*p* < 0.001), and acidity (*p* < 0.05), but not for bacterial count. Reduced-fat matrices yielded higher hydrolysis than full-fat. The inoculation × time interaction was significant (*p* < 0.001) for all variables; fat type × time reached *p* < 0.001 for hydrolysis and protein concentration, and *p* < 0.05 for pH and bacterial count. The three-way interaction was not significant for any variable.

When examining the three-way interaction means (Figure 2), the patterns split clearly between inoculated and uninoculated groups as time progressed. In inoculated samples, pH fell progressively; by 72 h it was 4.42 in full-fat with *L. plantarum* and 4.28 in reduced-fat with *L. plantarum*. Uninoculated samples stayed flat (6.59–6.72). Titratable acidity increased 5.8-fold in reduced-fat with *L. plantarum* (1.04% at 72 h) vs. 5.4-fold in full-fat; uninoculated remained ~0.2%. Degree of hydrolysis reached 61.3% (reduced-fat) and 52.8% (full-fat) with *L. plantarum* at 72 h, vs. < 4.1% in controls. Protein concentration declined only with inoculation: 4.21–4.33 mg/mL at 72 h vs. 5.75–5.76 in controls. *Lactiplantibacillus* count rose to 9.21 (full-fat) and 9.45 (reduced-fat) log10 CFU/mL by 72 h; undetectable without inoculation. Thus, longer fermentation with inoculation, especially in reduced-fat, correlated with lower pH and protein, and higher acidity, hydrolysis, and bacterial counts.

**Figure 2:**
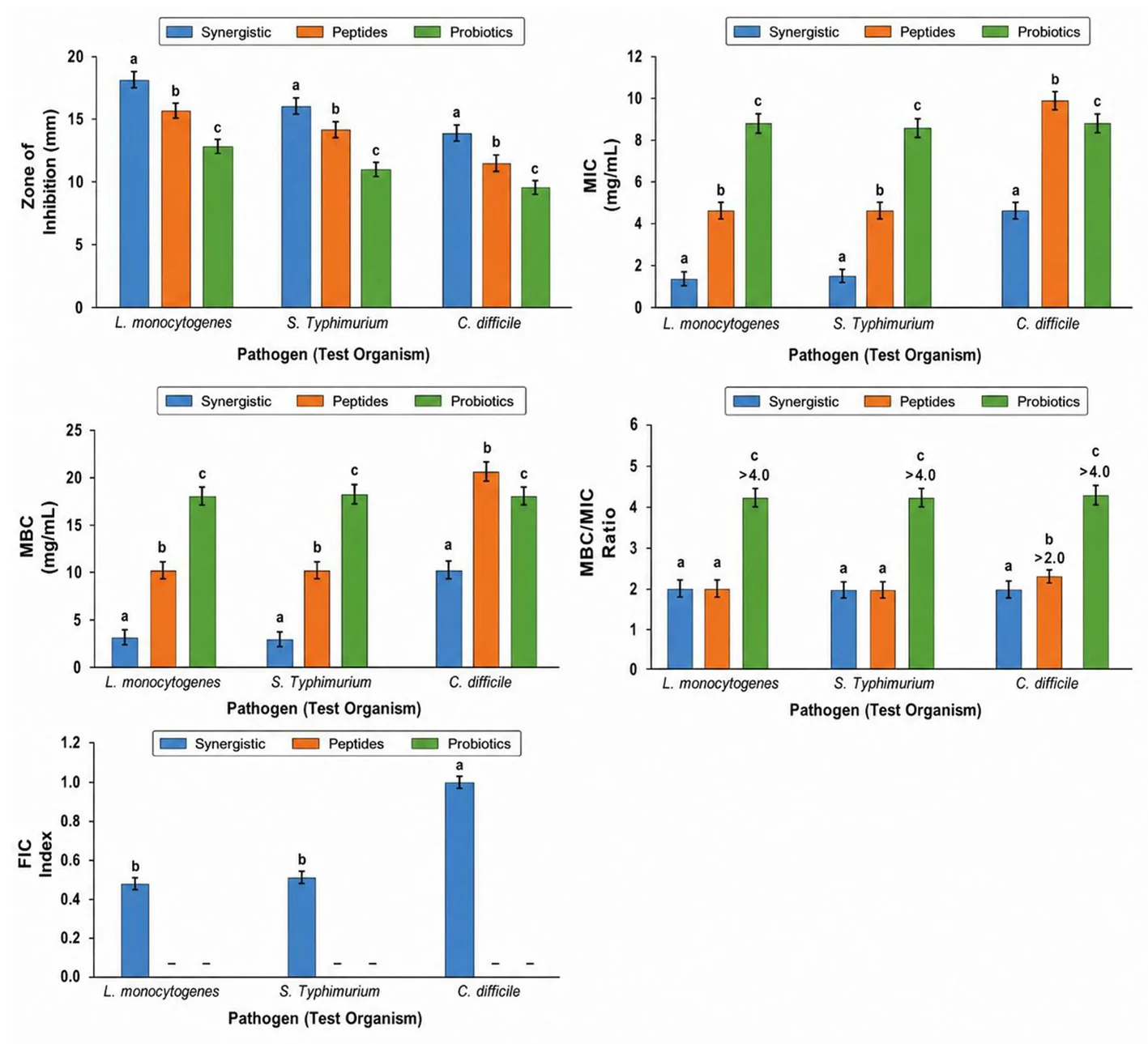
Three-way interaction effects of fat type (%), inoculation, and fermentation time (h) on pH (pH unit), titratable acidity (% lactic acid), degree of hydrolysis (%), protein concentration (mg/mL), and Lactiplantibacillus count (log_10_ CFU/mL) during Phase 1 fermentation. Values are mean ± SE, *n* = 3. ND = not detected. Within each variable, different lowercase letters above bars indicate statistically significant differences (*p* < 0.05) according to Tukey's HSD test across all 16 interaction means.

### Antimicrobial activity

The two-way ANOVA (Supplementary Table S2) explained 94.5–96.2% of the variance in antimicrobial outputs (R^2^ = 0.945 for zone of inhibition, 0.962 for MIC, 0.958 for MBC). Treatment type showed highly significant main effects (*p* < 0.001) on zone of inhibition, MIC, and MBC. Pathogen type was also significant (*p* < 0.001), and the treatment × pathogen interaction was significant (*p* < 0.001), indicating that treatment effects varied by pathogen. The FIC index was not included in the model because it applies only to the combination treatment.

Antimicrobial performance shifted with both treatment type and pathogen type (Figure 3). The combination consistently outperformed the individual treatments. Zone of inhibition for the synergistic mix reached 17.8 mm against *L. monocytogenes*, 16.1 mm against *S. Typhimurium*, and 14.2 mm against *C. difficile*, exceeding peptides or probiotics alone. Minimum inhibitory concentration (MIC) was lowest in the combination: 1.25 mg/mL for *L. monocytogenes*, 1.50 mg/mL for *S. Typhimurium*, and 5.00 mg/mL for *C. difficile*, a 75% reduction vs. peptides alone for the first two, and 50% for *C. difficile*. Probiotics alone gave the highest MIC across the panel. Minimum bactericidal concentration (MBC) mirrored the same trend, with the synergistic treatment at 2.5, 3.0, and 10.0 mg/mL, respectively; the MBC/MIC ratio held at 2.0 for all three, indicating bactericidal action. The fractional inhibitory concentration (FIC) index was 0.50 for *L. monocytogenes* (synergy), 0.53 for *S. Typhimurium* and 1.00 for *C. difficile* (both additivity). Overall, 5 mg/mL peptides plus 4.5 × 10^4^ CFU/mL probiotics aligned with larger zones and lower inhibitory and bactericidal concentrations across pathogens.

**Figure 3:**
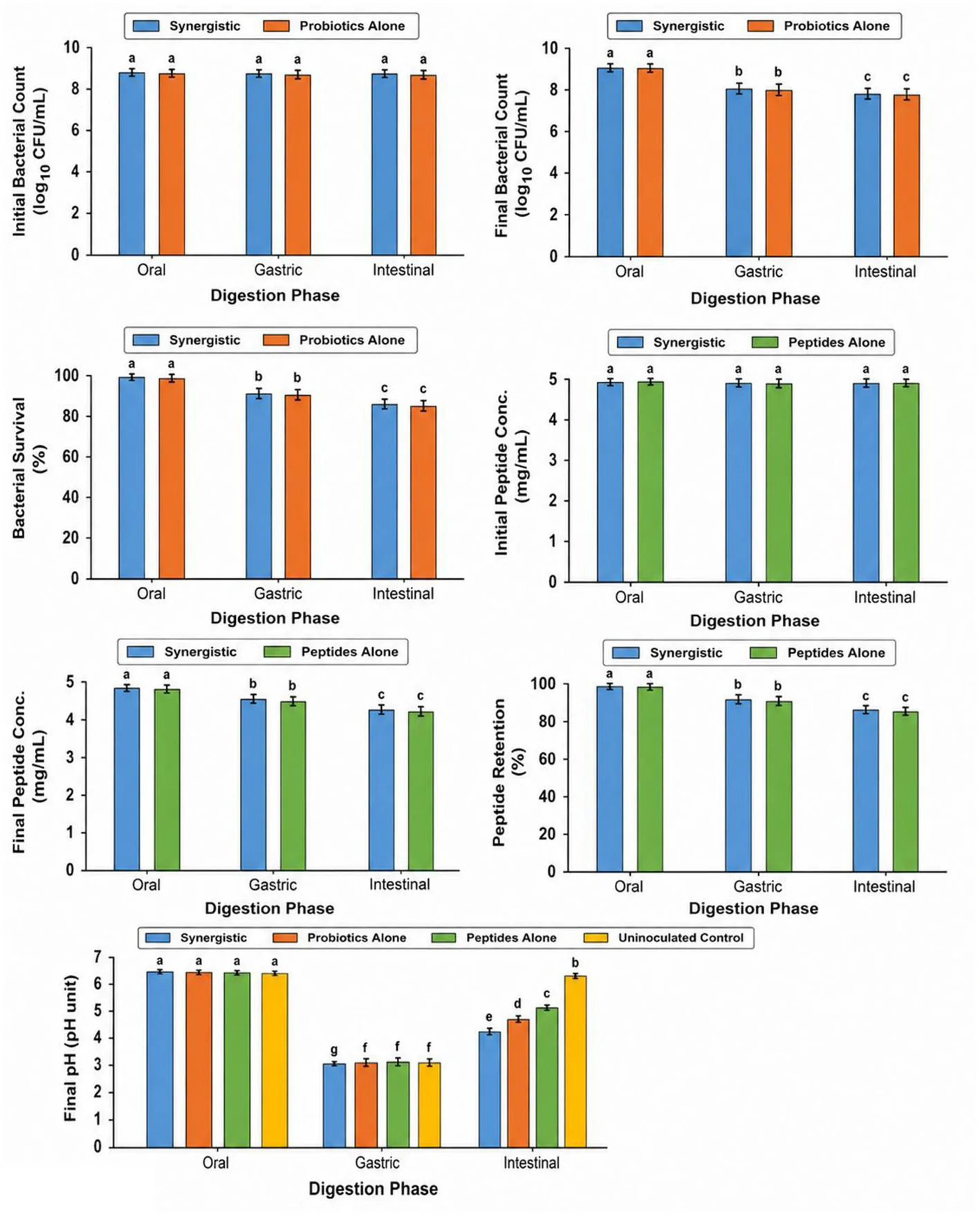
Quantitative antimicrobial parameters for effects of pathogen type and treatment on zone of inhibition (mm), minimum inhibitory concentration (mg/mL), minimum bactericidal concentration (mg/mL), minimum bactericidal concentration to minimum inhibitory concentration ratio, and fractional inhibitory concentration (FIC) index. Values are mean ± SE, *n* = 3. — indicates not applicable. Within each pathogen group and variable, different lowercase letters above bars indicate statistically significant differences (*p* < 0.05) according to Tukey's HSD test. FIC index ≤ 0.5 = synergy; >0.5 to ≤ 1.0 = additivity.

### Gastrointestinal stability

The two-way ANOVA (Supplementary Table S3) showed that treatment and digestion phase together explained 98.1–99.2% of the variation (R^2^ = 0.981–0.992) for final bacterial count, survival, final peptide, retention, and final pH. Both main effects and their interaction were highly significant (*p* < 0.001). Initial bacterial count and initial peptide concentration were fixed and therefore excluded from the model.

Moving from oral to gastric to intestinal phases pulled most metrics down, yet the combination treatment held up best (Figure 4). Final bacterial count fell to 7.84 and 7.76 log_10_ CFU/mL in the intestine for combination treatment and probiotics alone, down from 8.96 and 8.95 in the oral phase; bacterial survival followed, landing at 87.1% and 86.2% vs. 99.6% and 99.4%. For peptides, final peptide concentration in the intestine was 4.29 and 4.20 mg/mL, with peptide retention at 85.8% and 84.0%; in the oral phase retention was 99.6% and 99.0%. Final pH dipped lowest in gastric conditions, 3.05 to 3.15, then rebounded in the intestine to 4.42 and 5.41 in the active treatments, while the control reached 7.02. In short, digestion phase reduced counts, survival, peptide levels and retention, and shifted pH in two steps; the combination consistently retained more than either single treatment.

**Figure 4:**
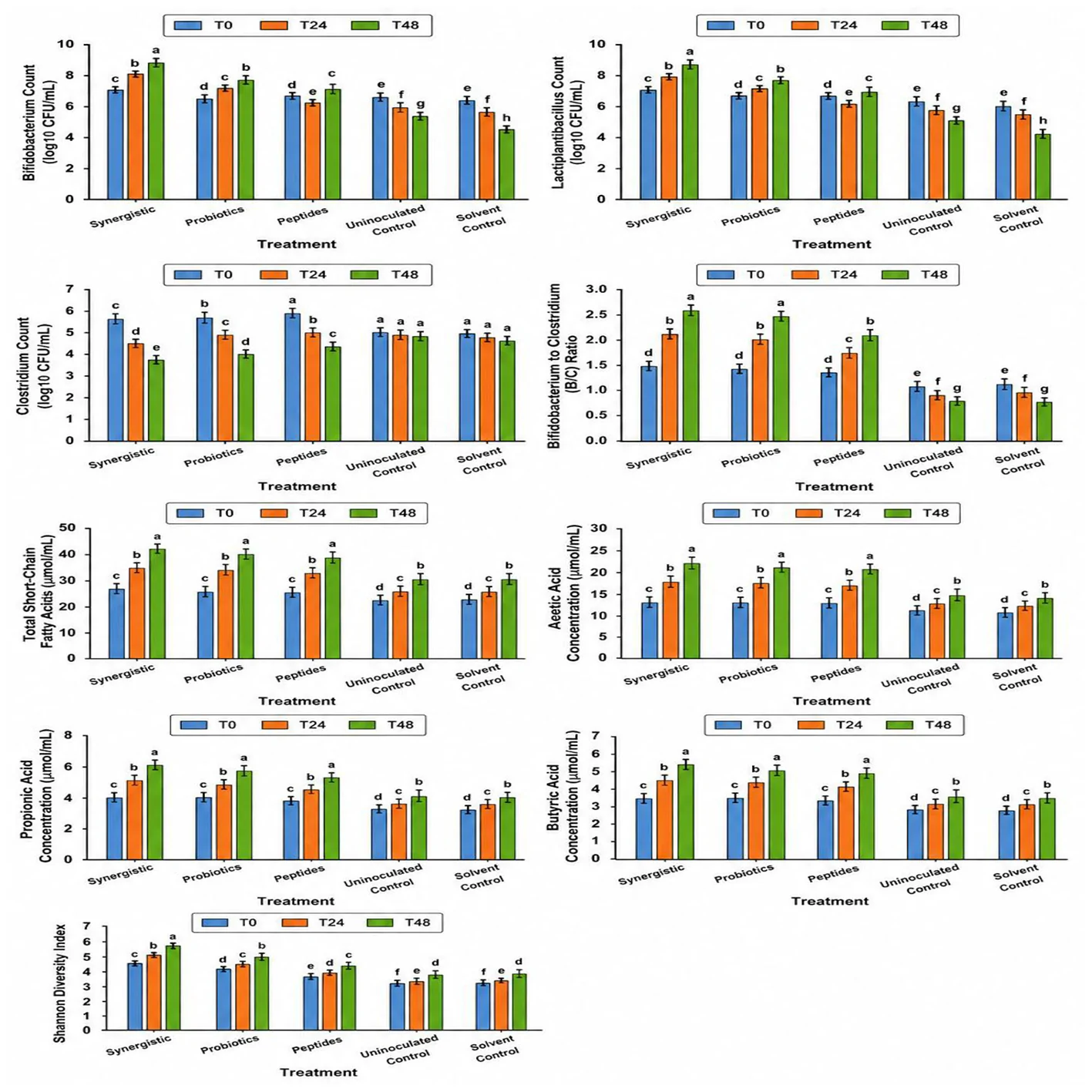
Treatment × digestion phase interaction effects of treatment and digestion phase on initial bacterial count (log_10_ CFU/mL), final bacterial count (log_10_ CFU/mL), bacterial survival (%), initial peptide (mg/mL), final peptide (mg/mL), peptide retention (%), and final pH (pH unit) during Phase 3A gastrointestinal digestion. Values are mean ± SE, *n* = 3. ND = not detected. Within each variable, different lowercase letters above bars indicate statistically significant differences (*p* < 0.05) according to Tukey's HSD test across all 12 interaction means. Bacterial survival (%) = (Final bacterial count / Initial bacterial count) × 100. Peptide retention (%) = (Final peptide concentration / Initial peptide concentration) × 100.

### Microbiota modulation

The two-way ANOVA (Supplementary Table S4) showed that treatment and fermentation time accounted for 98.5–99.7% of the variance (R^2^ = 0.985 for Shannon index to 0.997 for total SCFAs). Both main effects were significant (*p* < 0.001) for all nine outcomes: *Bifidobacterium* count, *Lactiplantibacillus* count, *Clostridium* count, B/C ratio, total SCFAs, acetic acid, propionic acid, butyric acid, and Shannon index. The interaction was also significant (*p* < 0.001), indicating that treatment differences varied depending on fermentation duration.

Over 48 h the combination treatment drove the clearest shifts (Figure 5). *Bifidobacterium* and *Lactiplantibacillus* counts rose in combination treatment and probiotics groups, reaching 8.93 and 8.71 log_10_ CFU/mL at T48 in the combination, about 14% above baseline, while controls fell to ~6.1 and ~5.5 log_10_ CFU/mL. *Clostridium* dropped, bottoming at 3.31 log_10_ CFU/mL in the combination treatment *vs*. ~7.1 in controls; accordingly, the B/C ratio climbed to 2.70.

**Figure 5:**
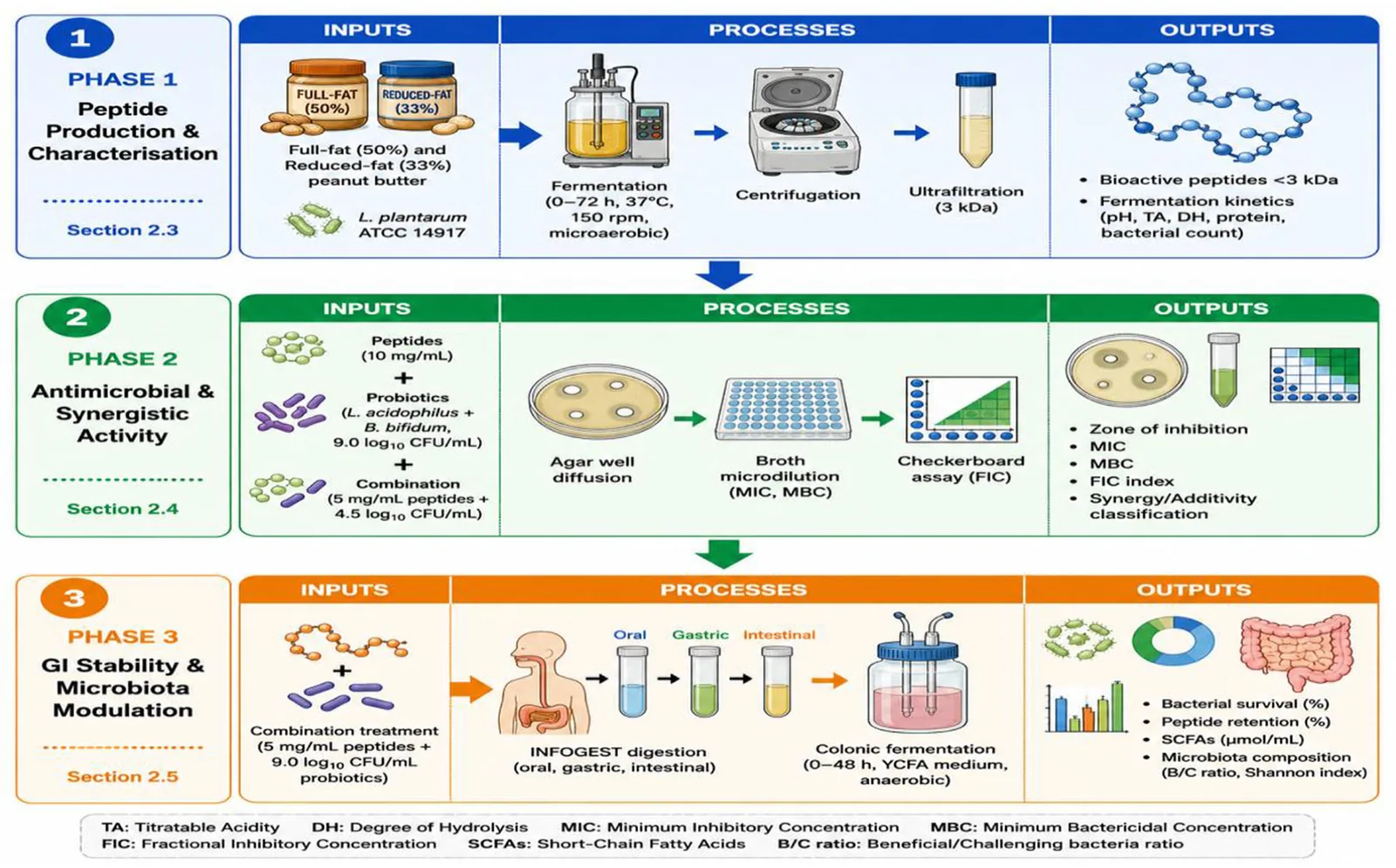
Treatment × fermentation time interaction effects of treatment and fermentation time (h) on *Bifidobacterium* (log_10_ CFU/mL), *Lactiplantibacillus* (log_10_ CFU/mL), *Clostridium* (log_10_ CFU/mL), B/C ratio, total short-chain fatty acids (μmol/mL), acetic acid (μmol/mL), propionic acid (μmol/mL), butyric acid (μmol/mL), and Shannon index during Phase 3B microbiota modulation. Values are mean ± SE, *n* = 3. ND = not detected. T0 = 0 h, T24 = 24 h, T48 = 48 h. B/C ratio = *Bifidobacterium* / *Clostridium*. Within each variable, different lowercase letters above bars indicate statistically significant differences (*p* < 0.05) according to Tukey's HSD test across all 15 interaction means.

Short-chain fatty acids tracked the same pattern. At T48 the combination treatment produced 46.5 μmol/mL total SCFAs, 30.5 μmol/mL acetic acid, 9.2 μmol/mL propionic acid, and 6.6 μmol/mL butyric acid, which are 5-6-fold above T0. Probiotics and peptides were intermediate; controls stayed below 20.8 μmol/mL. Shannon index also increased, to 4.39, 4.25, and 4.18 in combination treatment, probiotics, and peptides, vs. ~3.9 in controls. No growth or metabolites appeared in media control. Overall, longer fermentation with the combination or probiotics raised beneficial bacteria, the B/C ratio, all SCFA fractions, and diversity, while suppressing *Clostridium*.

## Discussion

### Phase 1: fermentation kinetics and peptide generation

From a therapeutic nutrition perspective, both fermentation time and *Lactiplantibacillus plantarum* inoculation emerged as the key drivers of acidification and proteolysis. The significant inoculation × time interaction for degree of hydrolysis (MS = 887.4, *p* < 0.001) suggests that peptide generation was metabolically driven, not simply a function of time. This aligns with Peres Fabbri et al. (39), who linked bioactive peptide release in fermented foods directly to microbial activity. In the same vein, Algboory and Muhialdin (6) reported that antimicrobial peptides from camel milk fermented with *L. plantarum* IS10 increased alongside bacterial growth. The progressive acidification (pH drop from 6.71 to 4.28) and concurrent rise in titratable acidity (from 0.18% to 1.04%) indicate active organic acid production, which not only contributes to antimicrobial effects but also enhances proteolytic enzyme activity by creating an optimal pH environment for *L. plantarum* proteases (40).

The reduced-fat matrix outperformed the full-fat matrix at 72 h, with higher hydrolysis (61.3% vs 52.8%), greater bacterial density (9.45 vs. 9.21 log_10_ CFU/mL), and more protein depletion (27.7% vs. 25.5%). We attribute this to reduced lipid interference and improved enzyme access to the protein substrate. Consistent with this, Christensen et al. (40) described how lipids can limit protease access to plant proteins by forming physical barriers, while Pi et al. (41) observed enhanced hydrolysis in pretreated peanuts fermented with *Bacillus natto* and *L. plantarum*. The higher carbohydrate content of the reduced-fat matrix (34.0% vs. 20.0%) likely provided a more readily fermentable substrate, supporting faster microbial growth and higher final cell densities, as *L. plantarum* preferentially metabolizes sugars for energy and acid production (42).

Interestingly, *L. plantarum* reached higher counts in the reduced-fat system. Although Treven et al. (12) found no effect of fat type on *Lactococcus* and *Streptococcus* during digestion, our data suggest that during fermentation, matrix composition may influence growth through improved nutrient diffusion, reduced cell entrapment in lipid aggregates, and enhanced oxygen transfer even under microaerobic conditions. The lower lipid content may also reduce the physical entrapment of bacterial cells within fat globules, allowing better access to nutrients and more homogeneous distribution throughout the medium (43). This mechanistic distinction between fermentation and digestion phases is important, as it highlights that matrix effects are context-dependent and may not translate directly across different physiological stages.

The non-significant three-way interaction indicates additive rather than synergistic effects among fat type, inoculation, and time. This differs from Oyedoh et al. (44), who reported synergy in peanut and cowpea fermentation by *Lactiplantibacillus* spp. Such discrepancies likely reflect differences in substrate protein structure, lipid distribution, and the specific strains used, which ultimately shape fermentation outcomes. The additive nature suggests that each factor independently contributes to the observed effects, simplifying process optimisation by allowing separate adjustment of each parameter.

### Phase 2: antimicrobial activity and synergy

Treatment type accounted for most of the variation in zone of inhibition, MIC, and MBC, with all effects significant at *p* < 0.001 and R^2^ values above 0.945. Crucially, the combination of 5 mg/mL peptides plus 4.5 × 104 CFU/mL probiotics reduced the MIC against *L. monocytogenes* by 75% (from 5.00 to 1.25 mg/mL) and produced an FIC index of 0.50. This meets the strict synergy criterion defined by Odds (33). This level of potentiation parallels Algboory and Muhialdin (6), who reported enhanced antimicrobial activity of *L. plantarum***-**derived camel milk peptides against foodborne pathogens and attributed it to complementary membrane-disruptive actions. The peptide fraction likely contains multiple cationic and amphipathic sequences that interact with the negatively charged bacterial membrane, causing permeabilisation and leakage of intracellular contents, while the probiotic at sub-inhibitory concentration may produce additional antimicrobial metabolites such as organic acids, hydrogen peroxide, and bacteriocins that weaken the pathogen's defenses (45).

In addition, the MBC:MIC ratio of 2.0 for all peptide-containing treatments confirms bactericidal activity, consistent with the criteria of Wiegand et al. (34). By contrast, probiotic-only treatments showed ratios above 4, indicating a purely bacteriostatic effect at the tested doses. Mechanistically, this suggests that peptides provided the lethal effect even at lower concentrations, while probiotics enhanced that effect without direct killing. Hernandez-Mendoza et al. (46) reported a similar pattern for bacteriocin-like substances from *Enterococcus lactis*, which inhibited growth but required higher doses for lethality. The bactericidal nature of the combination is clinically significant, as it suggests potential for use in food preservation and infection control where complete pathogen elimination is required rather than mere growth suppression.

Moreover, the pathogen-specific response, synergy against *L. monocytogenes* vs. additivity against *S. Typhimurium* and *C. difficile*, likely reflects differences in cell envelope structure. Amiri et al. (7) noted that the αs1-165-181 peptide was more active against Gram-positive than Gram-negative bacteria due to outer membrane barriers. Similarly, Festa et al. (47) found antimicrobial peptide 1018-K6 was more effective against *Salmonella enterica* than against spore-formers, which supports the weaker synergy observed here against *C. difficile*. The spore-forming nature of *C. difficile* and its ability to survive in a metabolically dormant state may also contribute to its reduced susceptibility, as antimicrobial peptides often target actively dividing cells (48). This pathogen-dependent efficacy suggests that the combination may be most effective against vegetative Gram-positive pathogens, with potential applications in targeting *Listeria* contamination in ready-to-eat foods.

### Phase 3A: gastrointestinal stability

From a clinical nutrition perspective, the digestion phase emerged as the key determinant of bacterial survival, peptide retention, and terminal pH, and notably the gastric phase drove most of the observed variance. The combination formulation retained 87.1% bacterial survival and 85.8% peptide retention at the intestinal endpoint, which was 0.9 percentage points higher than probiotics alone and 1.8 points higher than peptides alone. This protective pattern aligns with Saiz-Gonzalo et al. (13), who reported that pea protein microencapsulation improved probiotic survival during simulated gastrointestinal digestion by forming a matrix that buffers against acid and bile exposure. The retention advantage, though numerically modest, is biologically meaningful as it represents enhanced deliverability of both active components to the colon, where they can exert their microbiota-modulating effects.

In parallel, Yao et al. (15) demonstrated that peach gum polysaccharide incorporated into soy protein microparticles enhanced probiotic survival under INFOGEST conditions, which supports the notion that peptide-protein matrices can attenuate gastric stress. We observed a concurrent decline in viable counts from 8.96 to 7.84 log10 CFU/mL and in peptide concentration from 4.98 to 4.29 mg/mL across digestion phases, suggesting coupled stability; the peptide matrix likely provided a physical or nutritional shield, while metabolically active probiotics may have helped moderate local pH and reduce proteolysis through competitive effects. The peptide fraction may also act as a prebiotic substrate, supporting probiotic survival by providing fermentable nitrogen sources during gastric transit (30).

Treven et al. (12) showed that food matrix composition markedly influenced probiotic survival during gastrointestinal simulation, with protein-based matrices conferring superior protection relative to aqueous systems. Afzaal et al. (14) further reported greater survival for encapsulated vs. free cells under simulated gastrointestinal conditions. Importantly, the combination maintained viability at pH 3.05 during the gastric phase without disproportionate losses, which supports this mechanism and confirms that deliverability to the intestinal compartment was preserved. This resilience is critical for oral delivery, as it ensures that a sufficient fraction of viable probiotics and intact peptides reach the colon to exert their beneficial effects on gut microbiota and SCFA production.

### Phase 3B: microbiota modulation and SCFA production

Fermentation time was the primary driver of microbial succession and metabolite accumulation; however, the significant treatment × time interaction (*p* < 0.001) indicates that formulation dictated the trajectory of change. At 48 h, the combination yielded peak values for *Bifidobacterium* (8.93 log10 CFU/mL), *Lactiplantibacillus* (8.71), B/C ratio (2.70), total short-chain fatty acids (SCFAs) (46.5 μmol/mL), and Shannon index (4.39), while *Clostridium* declined to 3.31 log_10_ CFU/mL. This shift from proteolytic to saccharolytic metabolism mirrors Dong et al. (19), who linked probiotic modulation of the gut microbiota to attenuated inflammation via enrichment of beneficial taxa and suppression of *Clostridium*. The B/C ratio elevation from 1.13 to 2.70 is particularly noteworthy, as Magne et al. (49) identified this ratio as a relevant marker of gut dysbiosis, with higher values indicating a healthier, more saccharolytic ecosystem.

The 467% increase in total SCFAs, with butyric acid rising from 1.1 to 6.6 μmol/mL, is clinically meaningful; butyrate functions as a primary energy substrate for colonocytes, reinforces epithelial barrier integrity, and exerts anti-inflammatory effects through histone deacetylase inhibition. This aligns with Ashaolu et al. (4), who reviewed that saccharolytic fermentation by beneficial bacteria generates SCFAs that lower luminal pH and inhibit proteolytic taxa. Peterson et al. (16) also reported that SCFAs favor fermentative over proteolytic pathways. Cele et al. (18) demonstrated that probiotic-fermented beverages from bambara groundnut and cowpea sprouts modulated the gut microbiota and increased SCFAs, which reinforces the prebiotic-like effect observed here. The substantial butyrate increase is particularly significant, as butyrate deficiency has been implicated in inflammatory bowel disease and colorectal cancer risk (50).

Moreover, the 14% increase in Shannon index at T48 for the combination group, compared with minimal or negative trends in controls, suggests enhanced community evenness and resilience. Magne et al. (49) identified the B/C ratio as a marker of gut dysbiosis, while Tsai et al. (51) associated Firmicutes/Bacteroidetes ratio shifts with functional changes in inflammatory bowel disease. Haneishi et al. (50) further emphasized connections between microbiota composition and inflammatory conditions. The Shannon index improvement indicates that the combination not only enriched specific beneficial taxa but also increased overall microbial diversity, which is associated with greater resistance to pathogen colonization and metabolic flexibility (52).

The combination treatment outperformed probiotics or peptides alone, consistent with Éliás et al. (52), who in a systematic review and meta-analysis found greater microbiota diversity changes with combined probiotic interventions than with single-strain supplementation, implying that a synbiotic approach embedded in a food matrix is needed for substantial ecosystem reconfiguration. Our data also align with Reffai and Fechtali (20) on *Lactiplantibacillus* in Type II sourdough enhancing protein digestibility and bioactive peptide release. They further resonate with Staudacher and Whelan (21) on low-FODMAP fermented foods reducing osmotic stress while promoting saccharolytic fermentation. The techno-functional properties and biosafety of our peptide-probiotic combination meet criteria proposed by Marco et al. (22) for novel fermented starters. Finally, Oyedoh et al. (44) and Cele et al. (18) showed that *Lactobacillus*-matrix interactions in fermented beverages affect metabolic profiles, supporting the impact of the reduced-fat matrix observed here. The enhanced performance of the reduced-fat matrix in colonic fermentation may be attributed to its higher peptide content and lower lipid load, which reduces competition for fermentable substrates and favors saccharolytic bacteria over proteolytic ones (4).

### Limitations

Despite the strong *in vitro* data, several gaps still need to be addressed before we can claim clinical relevance. The synergy and bactericidal activity seen in agar and broth assays are promising; however, they do not automatically guarantee *in vivo* performance. As a result, animal models and human clinical trials are essential to test whether the 87.1% survival observed in the INFOGEST model actually translates into intestinal colonization and sustained bioactivity. The INFOGEST model, while standardized, cannot fully replicate the complex interactions of the human gastrointestinal tract, including peristalsis, mucus layer, immune cells, and resident microbiota, which may affect the stability and efficacy of the formulation (11, 36).

Another key unknown is the chemistry behind the bioactivity. The specific peptide sequences within the < 3 kDa fraction that drove the 467% surge in total SCFAs and the 52.4% suppression of *Clostridium* have not been identified. Therefore, proteomic sequencing (e.g., LC-MS/MS) is needed to map structure-function relationships and to pinpoint which peptides are responsible for the observed antimicrobial and prebiotic effects. Identification of active sequences would enable targeted production, optimisation, and potential commercialization of specific peptide-based ingredients.

Finally, our fermentation window was limited. The current evaluation ran for only 48 h of colonic fermentation, so we do not know whether the microbial community and metabolite pool remain stable over longer periods. Extended kinetic studies are required to confirm if the B/C ratio of 2.70 and Shannon index of 4.39 are maintained under chronic exposure, and to rule out drift or adaptation that could alter the health-promoting profile. Longer-term studies (e.g., 7-day continuous fermentation models) would also allow assessment of whether the observed shifts represent permanent reconfiguration or transient changes that revert upon cessation of treatment (23). Additionally, the use of fecal inocula from only three healthy donors, while reducing inter-individual variation, limits the generalisability of the findings; future studies should include a larger and more diverse donor cohort, including individuals with dysbiosis or metabolic disorders, to evaluate the formulation's efficacy across different gut ecosystems.

## Conclusions

This study shows that fermenting a reduced-fat peanut matrix with *Lactiplantibacillus plantarum* and then combining it with probiotics creates a single system that addresses antimicrobial activity, digestive survival, and gut microbiota modulation together. After 72 h of fermentation, we obtained 61.3% degree of hydrolysis and 9.45 log_10_ CFU/mL, and therefore the reduced-fat matrix can be considered a strong substrate for producing bioactive peptides.

In addition, the combination of 5 mg/mL peptides with 4.5 × 10^4^ CFU/mL probiotics achieved an FIC index of 0.50 against *Listeria monocytogenes* and maintained 87.1% bacterial survival and 85.8% peptide retention through simulated digestion. As a result, colonic fermentation improved markedly because *Bifidobacterium* and *Lactobacillus* increased, *Clostridium* decreased to 3.31 log_10_ CFU/mL, and total SCFAs rose by 467% to 46.5 μmol/mL.

Based on these findings, several recommendations emerge. Because the treatment × time interaction was very strong at R^2^ = 0.985–0.997, future work should fix fermentation at 72 h and follow the INFOGEST protocol closely to reproduce the 61.3% hydrolysis and 9.45 log_10_ CFU/mL results. Moreover, proteomic sequencing of the < 3 kDa fraction is needed to identify the specific peptides that drove the SCFA increase and *Clostridium* suppression, since this will support more targeted product development. At the same time, animal models and human trials are essential to verify whether the 87.1% survival observed *in vitro* translates into colonization, sustained activity, and measurable health benefits. Finally, with a target dose of 5 mg/mL peptides plus 4.5 × 10^4^ CFU/mL probiotics, this reduced-fat peanut formulation could serve as a cost-effective synbiotic, although full safety testing and dose standardization based on MBC values of 2.5 to 10.0 mg/mL are still required.

## Data Availability

The original contributions presented in the study are included in the article/supplementary material, further inquiries can be directed to the corresponding author.
