# Supplementary material for: Lactiplantibacillus plantarum peptides and probiotics from peanut butter enhance antimicrobial activity and gut microbiota modulation
Source: Front Nutr. 2026 Sep 17;13:1927533. doi: 10.3389/fnut.2026.1927533 (PMC13627428; doi:10.3389/fnut.2026.1927533)
Supplement: Supplementary file 1 [file Table_1.docx]

Supplementary Table S1. Mean square for effects of fat type (%), inoculation, and fermentation time (h) on pH (pH unit), titratable acidity (% lactic acid), degree of hydrolysis (%), protein concentration (mg/mL), and *Lactiplantibacillus* count (log₁₀ CFU/mL) during Phase 1 fermentation.

| **Source of Variation** | **df** | **pH** | **Titratable Acidity** | **Degree of Hydrolysis** | **Protein Concentration** | ***Lactiplantibacillus* Count** |
| --- | --- | --- | --- | --- | --- | --- |
| Fat Type | 1 | 0.0824** | 0.0012* | 5.76** | 0.1245*** | 0.0214 |
| Inoculation | 1 | 74.63*** | 5.542*** | 2918.4*** | 6.712*** | 72.48*** |
| Fermentation Time | 3 | 48.16*** | 2.347*** | 2251.3*** | 4.431*** | 41.54*** |
| Fat Type × Inoculation | 1 | 0.0145 | 0.0002 | 0.58 | 0.0081* | 0.1083*** |
| Fat Type × Time | 3 | 0.0268* | 0.0004 | 23.08*** | 0.0108*** | 0.0355* |
| Inoculation × Time | 3 | 11.46*** | 0.550*** | 887.4*** | 2.008*** | 18.23*** |
| Fat Type × Inoculation × Time | 3 | 0.0111 | 0.0002 | 0.54 | 0.0027 | 0.0224 |
| Error | 32 | 0.0092 | 0.0003 | 0.7925 | 0.0013 | 0.0092 |
| **R²** | — | 0.978 | 0.981 | 0.975 | 0.979 | 0.982 |

*Values are mean squares. df = degrees of freedom. *p < 0.05,* *p < 0.01, ***p < 0.001. R² = coefficient of determination. — indicates not applicable.*

Supplementary Table S2. Mean square for effects of treatment type and pathogen type on zone of inhibition (mm), minimum inhibitory concentration (mg/mL), and minimum bactericidal concentration (mg/mL) for antimicrobial parameters in Phase 2.

| **Source of Variation** | **df** | **Zone of Inhibition (mm)** | **MIC (mg/mL)** | **MBC (mg/mL)** |
| --- | --- | --- | --- | --- |
| Treatment Type | 2 | 152.87*** | 18.452*** | 38.452*** |
| Pathogen Type | 2 | 89.34*** | 6.740*** | 12.890*** |
| Treatment × Pathogen | 4 | 5.88*** | 1.280*** | 2.104*** |
| Error | 18 | 1.080 | 0.892 | 1.892 |
| **R²** | — | 0.945 | 0.962 | 0.958 |

*Values are mean squares. df = degrees of freedom. ***p < 0.001. R² = coefficient of determination. — indicates not applicable.*

Supplementary Table S3. Mean square for effects of treatment and digestion phase on final bacterial count (log₁₀ CFU/mL), bacterial survival (%), final peptide (mg/mL), peptide retention (%), and final pH (pH unit) for gastrointestinal parameters in Phase 3A.

| **Source of Variation** | **df** | **Final Bacterial Count** | **Bacterial Survival (%)** | **Final Peptide** | **Peptide Retention (%)** | **Final pH** |
| --- | --- | --- | --- | --- | --- | --- |
| Treatment | 3 | 0.0085*** | 3245.08*** | 0.0061*** | 14.852*** | 1.853*** |
| Digestion Phase | 2 | 0.0152*** | 5678.43*** | 0.0123*** | 29.847*** | 4.889*** |
| Treatment × Phase | 6 | 0.0011*** | 414.27*** | 0.0008*** | 1.964*** | 0.370*** |
| Error | 24 | 0.0001 | 17.50 | 0.0001 | 0.104 | 0.012 |
| **R²** | — | 0.991 | 0.987 | 0.989 | 0.981 | 0.992 |

*Values are mean squares. df = degrees of freedom.  ***p < 0.001. R² = coefficient of determination. — indicates not applicable.*

Supplementary Table S4. Mean square for effects of treatment and fermentation time (h) on *Bifidobacterium* (log₁₀ CFU/mL), *Lactiplantibacillus* (log₁₀ CFU/mL), *Clostridium* (log₁₀ CFU/mL), B/C ratio, total short-chain fatty acids (μmol/mL), acetic acid (μmol/mL), propionic acid (μmol/mL), butyric acid (μmol/mL), and Shannon index for microbiota modulation parameters in Phase 3B.

| **Source of Variation** | **df** | ***Bifidobacterium*** | ***Lactiplantibacillus*** | ***Clostridium*** | **B/C Ratio** | **Total SCFAs** | **Acetic Acid** | **Propionic Acid** | **Butyric Acid** | **Shannon Index** |
| --- | --- | --- | --- | --- | --- | --- | --- | --- | --- | --- |
| Treatment | 5 | 5.695*** | 4.853*** | 8.581*** | 0.948*** | 361.418*** | 212.034*** | 16.408*** | 29.870*** | 0.583*** |
| Fermentation Time | 2 | 10.437*** | 8.923*** | 19.234*** | 1.741*** | 662.891*** | 389.115*** | 30.112*** | 54.812*** | 1.069*** |
| Treatment × Time | 10 | 1.261*** | 1.090*** | 2.342*** | 0.215*** | 80.124*** | 47.063*** | 3.643*** | 6.632*** | 0.129*** |
| Error | 36 | 0.036 | 0.029 | 0.056 | 0.007 | 3.681 | 2.162 | 0.167 | 0.304 | 0.001 |
| **R²** | — | 0.991 | 0.993 | 0.994 | 0.989 | 0.997 | 0.996 | 0.994 | 0.995 | 0.985 |

*Values are mean squares. df = degrees of freedom.  ***p < 0.001. R² = coefficient of determination. — indicates not applicable. B/C ratio = Bifidobacterium / Clostridium.*
